# Early effect of percutaneous coronary intervention of non-left anterior descending artery on coronary flow velocity reserve of left anterior descending artery assessed by transthoracic Doppler echocardiography

**DOI:** 10.1371/journal.pone.0256161

**Published:** 2021-08-13

**Authors:** Masahiro Hada, Masahiro Hoshino, Nobutaka Wakasa, Tomoyo Sugiyama, Yoshihisa Kanaji, Masao Yamaguchi, Toru Misawa, Tatsuhiro Nagamine, Kai Nogami, Yumi Yasui, Taishi Yonetsu, Tetsuo Sasano, Tsunekazu Kakuta

**Affiliations:** 1 Division of Cardiovascular Medicine, Tsuchiura Kyodo General Hospital, Tsuchiura, Ibaraki, Japan; 2 Department of Cardiovascular Medicine, Tokyo Medical and Dental University, Bunkyo, Tokyo, Japan; Universita degli Studi Magna Graecia di Catanzaro, ITALY

## Abstract

**Background:**

Limited data are available regarding the influence of percutaneous coronary intervention (PCI) of non-totally occluded lesions (non-CTO) on the coronary flow of non-target vessels. We sought to investigate the short-term impact of the non-left anterior descending artery (non-LAD) PCI on the coronary flow physiology of LAD using transthoracic Doppler echocardiography (TDE).

**Methods and results:**

We consecutively studied 50 patients who underwent successful PCI of non-LAD and non-CTO lesions and a coronary flow velocity assessment of LAD at rest and maximal hyperemia before and at 2 days after the procedure by TDE. Coronary flow velocity reserve (CFVR) was calculated as the ratio of hyperemic to resting diastolic peak velocity (hDPV/bDPV). We evaluated the changes in LAD coronary flow characteristics after PCI of non-LAD and explored the determinants of the change in LAD-CFVR. The median fractional flow reserve (FFR) of the culprit lesion and the LAD quantitative flow ratio (QFR) were 0.67 and 0.88, respectively. After non-LAD PCI, LAD-CFVR was decreased in 33 patients (66.0%). LAD-CFVR significantly decreased (pre-PCI: 2.41, post-PCI: 2.03, p = 0.001) due to a significant decrease in LAD-hDPV (P = 0.007). The prevalence of impaired LAD-CFVR (≤2.0) significantly increased (pre: 30%, post: 48%, P = 0.027). Multivariable linear regression analysis showed that pre-PCI LAD-CFVR was independent predictor of the change in LAD-CFVR after PCI.

**Conclusions:**

LAD-CFVR significantly decreased after successful non-LAD PCI due to the postprocedural reduction of coronary flow assessed by LAD-hDPV.

## Introduction

The aim of revascularization by percutaneous coronary intervention (PCI) is to increase coronary blood flow in an ischemic region by modifying the epicardial lesion. A previous study using positron emission tomography (PET) showed a significant increase in regional stress myocardial blood flow (MBF) and myocardial flow reserve (MFR) in the region with revascularization, but no significant change in the regional stress MBF or MFR in remote territories without revascularization [1]. A recent study, in contrast, showed that stress MBF increased not only in chronic totally occluded lesions (CTO) but also in remote areas after PCI [2]. Previous studies demonstrated that successful PCI of an RCA-CTO resulted in a modest but significant increase in the fractional flow reserve (FFR) of the predominant donor vessel with a reduction in its coronary flow [3,4].

In contrast, limited data are available regarding the impact of PCI on non-CTO lesions without visible collaterals on the coronary blood flow physiology of remote non-target vessels.

Stress echocardiography has been a cost-effective modality providing diagnostic and prognostic information [5] on the coronary flow velocity reserve (CFVR) of a vessel. CFVR obtained by transthoracic Doppler echocardiographic (TDE) imaging during stress echocardiography can provide useful quantitative information on the functional status of coronary artery circulation [6–8]. TDE-CFVR has been extensively validated in the prognosis of patients with stable coronary artery disease [9–11], but the significance and possible role of CFVR evaluation in non-culprit vessels after PCI of non-CTO lesions have not been specifically investigated. TDE, when performed in the days after PCI, overcomes most of the limitations of the wire-based, invasive intracoronary pressure approach, such as under- or overestimation of CFR and FFR because of reactive resting hyperemia and microvascular injury [12–14]. Therefore, the two-fold aim of the present study was to (1) examine the early influence of PCI for non-LAD and non-CTO lesions on LAD coronary physiology by TDE and (2) explore the determinants of the change in LAD-CFVR.

## Materials and methods

The study protocol was approved by the ethics committee at Tsuchiura Kyodo General Hospital. The reference number is 918. All our patients provided written informed consent for the study and future data use.

### Study design and patient population

Consecutive patients who were scheduled for elective PCI for non-LAD and non-CTO lesions with anginal symptoms (Canadian Cardiovascular Society class 1–3) [15] were recruited prospectively in Tsuchiura Kyodo General Hospital between April 2020 and December 2020. All patients had functional significant stenosis in non-LAD lesion (positive noninvasive test results or invasive tests, including FFR ≤0.80, or positive resting indices). Patients with an acute coronary syndrome or angiographically visible collateral flow were excluded. Other exclusion criteria were the inability to provide consent, history of previous myocardial infarction, coronary intervention or coronary artery bypass graft, occluded target vessels, left main coronary artery disease, reduced systolic function (ejection fraction <50%), chronic renal disease, and contraindications to adenosine. Patients with a LAD lesion with more than 70% stenosis were not recruited. Of the 58 initially enrolled patients who underwent PCI and TDE before and after PCI (1 day before the procedure and 2 days after), 6 patients were excluded due to insufficient TDE data acquisition. Further, one patient was excluded because they showed a type 4A myocardial infarction (high-sensitivity cardiac troponin I greater than the upper reference limit of the 70 × 99^th^ percentile and associated ECG changes), and another was exempt due to consent withdrawal before completing the postprocedural TDE. Thus, the final analysis included 50 patients who underwent successful PCI and showed complete TDE flow data. Optimal medical therapy with high-dose statins, dual antiplatelets, and antihypertensives was initiated immediately after diagnostic catheterization in all patients. According to the study protocol, no *ad hoc* PCI was performed in the study patients.

Our study was approved by the institutional ethics committee (reference no. 918/Tsuchiura; April 3, 2020) and conducted in compliance with the tenets of the Declaration of Helsinki for investigation in human beings. All our patients provided written informed consent for the study and future data use.

### Invasive coronary angiography, percutaneous coronary intervention, and FFR measurements

Each patient initially underwent standard diagnostic coronary angiography via the radial artery using a 5F system to assess the coronary anatomy and severity of functional stenosis. Quantitative coronary angiography analyses were performed using a CMS-MEDIS system (Medis Medical Imaging Systems, Leiden, Netherlands). All patients received a bolus injection of heparin (5000 IU) before the procedure. Intracoronary bolus injections of nitroglycerin (0.2 mg) were administered at the start of the procedure and before functional measurements.

FFR was determined using a Radi Analyzer Xpress instrument with a PressureWire™ (Abbott Vascular, St. Paul, MN, USA). The details are in the Supplemental method.

Eligible patients who had non-totally occluded and non-LAD functionally significant stenosis with or without LAD stenosis (<70% stenosis) subsequently underwent preprocedural LAD-CFVR assessment before the scheduled PCI procedure for the non-LAD culprit lesion. PCI was performed using a 6F system via the radial artery and according to the latest guidelines [16]. The details are in the Supplemental method.

### LAD functional assessment using quantitative flow ratio (QFR)

Functional assessment of pre-PCI LAD was performed using the QFR. Computation of QFR was performed offline using proprietary software (QAngio XA 3D 2.0.28.0; Medis Medical Imaging Systems B.V., Leiden, Netherlands). A detailed description of the QFR measurement is presented in the supplementary materials.

### CT-derived cardiac mass analysis

Quantitative assessments of the LV mass were performed using the Aquarius iNtuition Workstation Edition version 4.4.13 (TeraRecon Inc., Foster City, CA, USA) at the mid-diastole phase as previously described [17–19]. The cardiac mass of the target lesion, the total mass of the target vessel, and total vessel mass of LAD were measured by two independent investigators (T.S. and Y.K.). A detailed description of CCTA acquisition and CCTA analysis is presented in the supplementary materials.

### Measurement of coronary flow velocity reserve

Eligible patients underwent pre- (one day before) and postprocedural (2 days after) LAD-CFVR assessments. Echocardiographic studies were conducted according to the American Society of Echocardiography guidelines using a commercially available digital ultrasound system (GE Vivid E95; GE Vingmed Ultrasound, Horten, Norway) with a multifrequency transducer and second-harmonic technology [20]. After a standard examination, coronary flow in the mid-distal portion of the LAD was searched in a modified three-chamber view. For color flow mapping, the velocity range was set as 16–24 cm/sec. A sample volume (3–5 mm wide) was positioned on the distal LAD to measure blood flow velocity. Peak diastolic coronary flow velocity was measured in basal conditions (bDPV) and during maximal hyperemia (hDPV), which was induced by intravenous adenosine (140 μg/kg per min through a central vein). All studies were digitally stored for offline review and measurements. Three optimal flow signal profiles at rest and during hyperemia were obtained offline from the recorded data. CFVR was calculated as the ratio of hyperemic to basal peak diastolic flow velocities using the software package of the ultrasound system. The averaged values obtained by the two observers were used for the final analysis.

**Fig 1:**
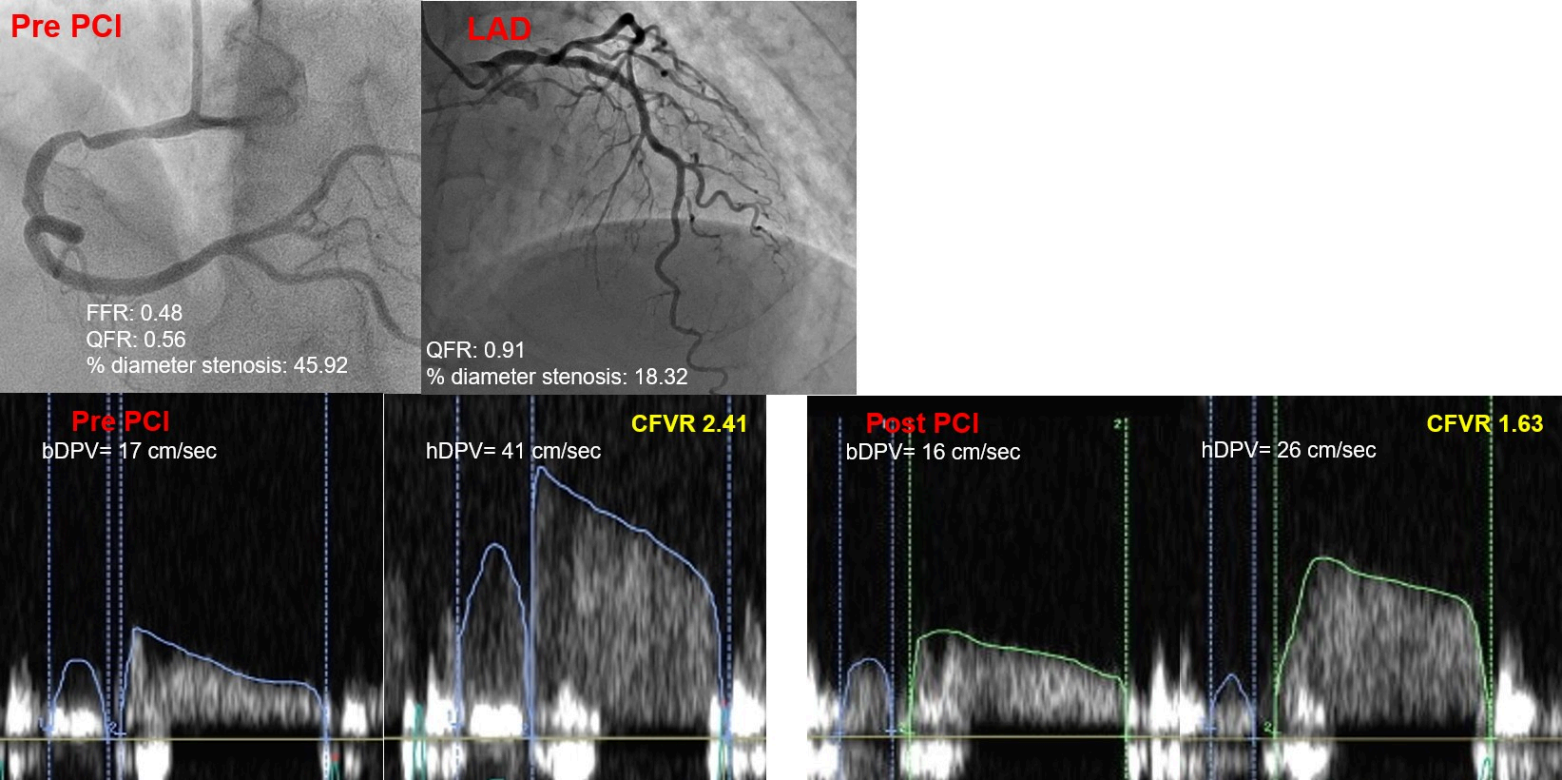
A representative case of a change in LAD-CFVR after non-LAD PCI by transthoracic Doppler echocardiography. An 80-year-old man without a significant stenosis in LAD. After successful RCA-PCI, LAD-CFVR decreased from 2.41 to 1.63 due to the reduction of hDPV. LAD, left anterior descending artery; CFVR, coronary flow velocity reserve; PCI, percutaneous coronary intervention; RCA, right coronary artery; hDPV, hyperemic peak diastolic velocity.

Two expert investigators for CFVR assessments, blinded to the clinical data, separately analyzed all stored data a week apart from each other and performed their analyses twice. Inter- and intra-observer agreements for the identification of impaired CFVR (≤2.0) were 94% and 96%, respectively. The mean difference in CFVR values between the two observers was 8.5%. All patients were instructed to refrain from consuming caffeine products at least 24 h before TDE stress echocardiography. Fig 1 shows a representative case of CFVR recording and measurement in the LAD before and after non-LAD lesion PCI.

### Statistical analysis

Statistical analyses were performed using the SPSS version 25.0 software (SPSS, Inc., Chicago, IL, USA). Categorical data were expressed as numbers and percentages and compared using the Chi-square or Fisher’s exact tests, as appropriate. The normality of the distributed values was assessed using Shapiro-Wilk statistics. Continuous data were expressed as median (interquartile range [IQR]) and analyzed using the Mann–Whitney U test with non-normal distribution. Comparisons between paired data were performed using the Wilcoxon’s signed-rank test or McNemar test, as appropriate. Associations were evaluated by analyzing the Pearson’s correlation for normally distributed data or Spearman’s correlation for non-normally distributed data. Univariable linear regression analysis was performed to identify significant predictors of the change in LAD-CFVR after PCI of a non-culprit lesion. Also, univariable logistic regression analyses were performed to predict a decrease in CFVR after PCI. Receiver operating characteristics (ROC) curve analysis was performed to assess the best cutoff values of pre-PCI LAD-CFVR, QFR, and diameter stenosis in LAD to predict a change in LAD-CFVR. The optimal cutoff value was calculated using the Youden index. A level of P<0.05 was considered significant.

## Results

### Baseline patient characteristics and physiological findings

**Fig 2:**
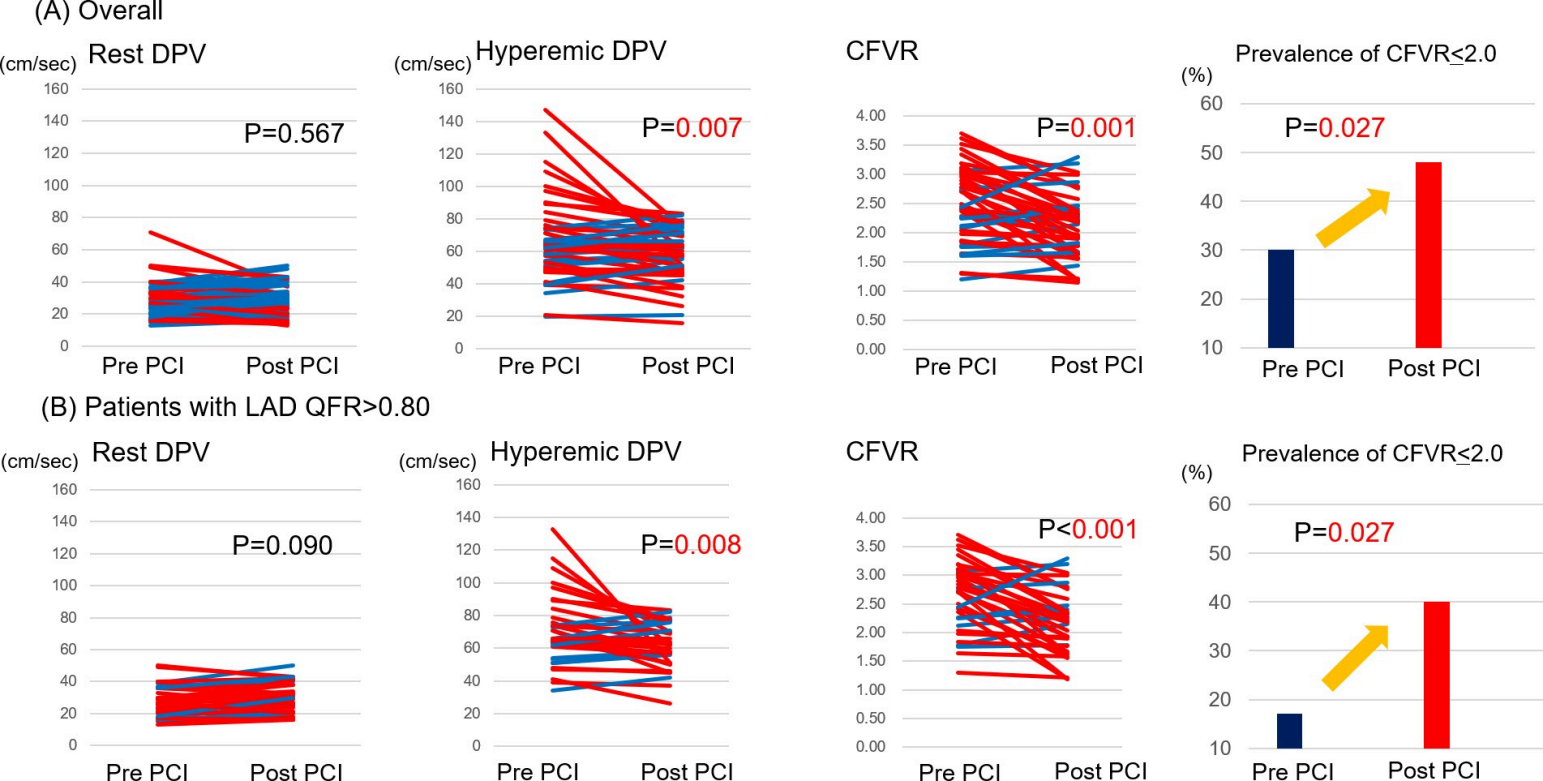
Changes in LAD-hDPV and LAD-CFVR after non-LAD PCI. After non-LAD PCI, LAD-hDPV and CFVR significantly decreased, whereas no significant change in LAD-bDPV was observed. (A) In a total cohort, the prevalence of LAD-CFVR of ≤2.0 after non-LAD PCI showed an increasing trend. (B) In the subgroup analysis of patients with functional less severe LAD stenosis (QFR>0.80), the prevalence of LAD-CFVR of ≤2.0 after non-LAD PCI was significantly increased. Abbreviations as in Fig 1.

Table 1 summarizes the baseline characteristics and angiographic and physiological data of 50 patients in two groups divided by the increase or decrease in LAD-CFVR after PCI. FFR values of the culprit lesion improved from 0.67 (0.53–0.76) to 0.92 (0.87–0.95). After successful PCI on the culprit lesion in 30 RCA-culprit and 20 LCx-culprit patients, LAD-hDPV (63.5–57.0 cm/s, P = 0.007) and CFVR (2.41–2.03, P = 0.001) significantly decreased, whereas no significant change was observed in LAD-bDPV (Fig 2). In total, 33 patients (66.0%) showed decreased LAD-CFVR after PCI. In patients with decreased LAD-CFVR after non-LAD culprit PCI, the angiographic diameter stenosis of LAD was milder and pre-PCI LAD-CFVR was higher compared with those showing increased LAD-CFVR.

**Table 1:** Baseline characteristics.

	Overall (N = 50)	LAD-CFVR decrease (N = 33)	LAD-CFVR increase (N = 17)	P-value
Age, years	69 ± 8	69 ± 8	69 ± 8	0.942
Male	44 (88.0)	29 (87.9)	15 (88.2)	1.000
Hypertension	37 (74.0)	26 (78.8)	11 (64.7)	0.322
Diabetes mellitus	20 (40.0)	13 (39.4)	7 (41.2)	1.000
Dyslipidemia	24 (48.0)	17 (51.5)	7 (41.2)	0.559
NT pro-BNP, pg/ml	178 (92–707)	160 (72–399)	291 (127–1467)	0.067
Lesion diameter stenosis, %	66.3 ± 14.3	66.2 ± 13.5	63.5 ± 16.0	0.546
Lesion diameter stenosis after PCI, %	15.1 (11.2–22.0)	15.3 (11.5–20.3)	14.4 (10.5–22.4)	0.700
LAD diameter stenosis, %	33.7 ± 13.4	30.7 ± 11.9	39.4 ± 14.8	**0.028**
AHA class type B2 or C	13 (26.0)	10 (30.3)	3 (17.6)	0.499
Ejection fraction, %	60 (54–69)	60 (50–69)	60 (58–69)	0.608
E/e’	12 (10–15)	13 (10–16)	12 (10–12)	0.305
Pre-PCI LAD-CFVR	2.41 (1.84–2.99)	2.73 (2.28–3.10)	1.82 (1.61–2.25)	**<0.001**
Pre-PCI LAD-basal DPV, cm/sec	26 (20–34)	26 (20–36)	27 (24–33)	0.845
Pre-PCI LAD-hyperemic DPV, cm/sec	64 (50–74)	66 (58–76)	50 (47–62)	**0.002**
Post-PCI LAD-CFVR	2.03 (1.67–2.45)	1.96 (1.63–2.31)	2.28 (1.78–2.57)	0.239
Post-PCI LAD-basal DPV, cm/sec	27 (21–33)	30 (25–38)	25 (19–28)	0.012
Post-PCI LAD-hyperemic DPV, cm/sec	57 (47–70)	62 (51–71)	49 (45–57)	0.043
Subtended target lesion cardiac mass, mm^3^	38.4 ± 14.5	39.6 ± 16.1	36.0 ± 10.6	0.454
PCI target vessel total mass, mm^3^	41.6 ± 15.4	43.0 ± 17.7	39.2 ± 10.3	0.470
LAD total vessel mass, mm^3^	66.4 ± 21.5	68.1 ± 18.6	63.2 ± 26.3	0.499
LAD QFR	0.88 (0.78–0.94)	0.90 (0.84–0.94)	0.83 (0.70–0.91)	**0.023**
Pre-PCI target lesion FFR	0.67 (0.53–0.76)	0.71 (0.52–0.78)	0.64 (0.53–0.71)	0.247
Post-PCI target lesion FFR	0.92 (0.87–0.95)	0.90 (0.84–0.94)	0.94 (0.93–0.96)	0.094
Pre-PCI target lesion QFR	0.66 (0.56–0.72)	0.65 (0.54–0.73)	0.66 (0.58–0.70)	0.712
Post-PCI target lesion QFR	0.92 (0.82–0.95)	0.91 (0.79–0.96)	0.93 (0.83–0.95)	0.623
Delta target lesion QFR	0.23 (0.17–0.37)	0.22 (0.15–0.38)	0.26 (0.22–0.34)	0.162
Pre-PCI basal systolic blood pressure, mmHg	130 (115–145)	127 (115–142)	133 (121–149)	0.712
Pre-PCI basal heart rate, /min	63 (57–69)	63 (56–69)	65 (58–69)	0.259
Pre-PCI hyperemic systolic blood pressure, mmHg	106 (95–123)	105 (93–121)	110 (104–133)	0.265
Pre-PCI hyperemic heart rate, /min	72 (66–79)	71 (66–79)	74 (68–77)	0.661
Post-PCI basal systolic blood pressure, mmHg	126 (114–137)	126 (115–137)	121 (107–146)	0.958
Post-PCI basal heart rate, /min	67 (59–72)	67 (61–70)	65 (59–74)	0.854
Post-PCI hyperemic systolic blood pressure, mmHg	109 (95–121)	104 (91–114)	119 (106–129)	0.080
Post-PCI hyperemic heart rate, /min	75 (69–82)	77 (68–82)	74 (69–80)	0.964

Data are presented as n (%) or median (Q1-Q3).

LAD = left anterior descending artery, AHA = American heart association, PCI = percutaneous coronary intervention, CFVR = coronary flow velocity reserve, DPV = diastolic peak velocity, QFR = quantitative flow ratio, FFR = fractional flow reserve.

Since vasodilators could affect coronary flow, we performed a subgroup analysis of the cohort in which 3 patients were excluded on the prescription of oral nitrates before and after PCI. The main result was consistent that LAD-CFVR significantly decreased after non-LAD PCI (pre-PCI 2.41 [1.85–2.98], post-PCI 2.03 [1.66–2.43], p = 0.002).

### Determinants of changes in post-PCI CFVR

**Fig 3:**
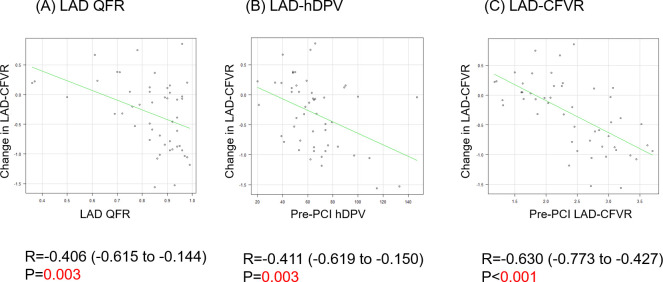
Correlation analyses between LAD angiographic and functional stenosis severity, and the change in LAD-CFVR. LAD-QFR values were correlated with the changes in LAD-CFVR (post-PCI CFVR–pre-PCI CFVR, A). Additionally, pre-PCI LAD-hDPV and pre-PCI LAD-CFVR were correlated with changes in LAD-CFVR (B and C). QFR: Quantitative flow ratio. Other abbreviations as in Fig 1.

The changes in LAD-CFVR were significantly associated with pre-PCI LAD functional stenosis severity assessed by QFR, indicating that milder LAD stenosis (higher QFR) resulted in a greater decrease in post-PCI LAD-hDPV and LAD-CFVR (Fig 3).

The results of univariable linear regression analyses to predict changes in CFVR after PCI are shown in Table 2. The results of univariable logistic analysis similarly revealed that pre-PCI LAD diameter stenosis, pre-PCI LAD functional stenosis severity, pre-PCI LAD-hDPV, and pre-PCI LAD-CFVR were significant predictors of a decrease in CFVR after PCI (Table 3). Multivariable linear regression analysis showed that pre-PCI LAD-CFVR was an independent predictor of the change in LAD-CFVR after PCI (Table 2).

**Table 2:** Linear regression analysis for the change in LAD-CFVR after non-LAD PCI.

	Univariable analysis			Multivariable analysis		
	β	95%CI	P value	β	95%CI	P value
Beta Blocker	0.215	-0.170–0.600	0.268			
NT pro-BNP, pg/ml	1.000	0.999–1.000	0.290			
LAD diameter stenosis, %	0.008	0.004–0.020	0.192			
E/e’	1.090	0.921–1.280	0.330			
Pre-PCI LAD-hyperemic DPV, cm/sec	-0.010	-0.016 to -0.003	**0.003**	-0.005	-0.010–0.001	0.107
Pre-PCI LAD-CFVR	-0.538	-0.743 to -0.346	**<0.001**	-0.473	-0.678 to -0.268	**<0.001**
LAD total vessel mass, mm^3^	0.0051	-0.0043–0.0145	0.278			
LAD QFR	-1.673	-2.706 to -0.568	**0.003**			
Pre-PCI target lesion QFR	-0.597	-1.663–0.469	0.266			

CI = confidence interval, LAD = left anterior descending artery, PCI = percutaneous coronary intervention, DPV = diastolic peak velocity, CFVR = coronary flow velocity reserve, QFR = quantitative flow ratio.

**Table 3:** Logistic regression analysis for the decrease in LAD-CFVR after non-LAD PCI.

	Odds ratio	95%CI	P value
RCA: PCI target vessel	0.327	0.088–1.21	0.095
LAD diameter stenosis, %	0.949	0.904–0.997	**0.036**
Pre-PCI LAD-hyperemic DPV, cm/sec	1.050	1.010–1.090	**0.019**
Pre-PCI LAD-CFVR	6.560	1.980–21.700	**0.002**
LAD QFR	323.0	2.200–47300	**0.023**

CI = confidence interval, RCA = Right coronary artery, PCI = percutaneous coronary intervention, LAD = left anterior descending artery, DPV = diastolic peak velocity, CFVR = coronary flow velocity reserve, QFR = quantitative flow ratio.

**Fig 4:**
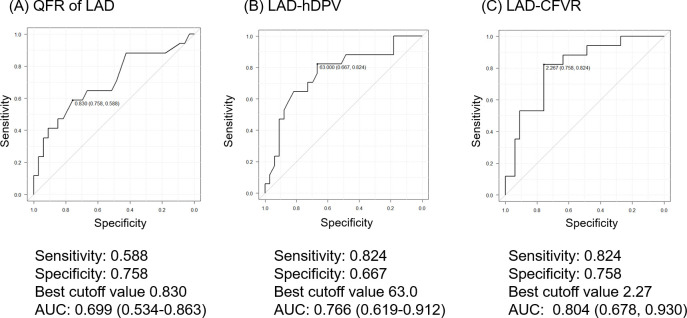
Diagnostic performance for improvement of LAD-CFVR. ROC curve analyses for (A) LAD-QFR, (B) pre-PCI LAD-hDPV, and (C) pre-PCI LAD-CFVR with the improvement of LAD-CFVR. Abbreviations as in Fig 1.

ROC analyses revealed the best cutoff values for CFVR increase after PCI as follows: LAD QFR = 0.83 (AUC: 0.699, 95%CI: 0.534–0.863, P = 0.003), pre-PCI LAD-hDPV = 63.0 (AUC: 0.766, 95%CI: 0.619–0.912, P = 0.003), and pre-PCI LAD-CFVR = 2.27 (AUC: 0.804, 95%CI: 0.678–0.930, P<0.001) (Fig 4).

The prevalence of LAD-CFVR ≤2.0 after non-LAD PCI showed a significant increase (pre-PCI: 30%, post-PCI: 48%, P = 0.027). In the subgroup analysis of patients with less severe functional LAD stenosis (QFR>0.80), the prevalence of LAD-CFVR ≤2.0 after non-LAD PCI showed a significant increase (pre-PCI: 17%, post-PCI: 40%, P = 0.027) (Fig 2).

## Discussion

The current study used TDE to investigate the early influence of non-CTO and non-LAD PCI on the coronary flow physiology of LAD. The major findings were: (1) LAD-CFVR decreased in more than half of the patients (66.0%) due to a significant decrease in LAD-hDPV; (2) Pre-PCI LAD-CFVR was an independent predictor of a change in CFVR; (3) The prevalence of LAD-CFVR ≤2.0 after non-LAD PCI showed a significant increase.

This study was the first to compare the coronary flow at rest and during vasodilator stress and depict it using TDE, where successful and uncomplicated non-CTO and non-LAD PCI significantly influenced both CFVR and LAD-hDPV when evaluated early after PCI.

The changes of LAD-CFVR and LAD-hDPV after non-LAD PCI affect pressure indices of LAD region. The decision to conduct LAD PCI may change according to the timing of LAD functional assessment, even though its exact effect on FFR remains to be determined.

### Coronary flow redistribution after revascularization in CTO lesions

Coronary flow redistribution after revascularization has been extensively investigated in RCA-CTO lesions, and the changes in the dominant collateral flow from the LAD after CTO-PCI have been reported [3,4,21–23]. These previous studies have demonstrated, in general, that there is a decrease in collateral blood supply to the myocardial region distal to the CTO after recanalization, an increase in FFR in the predominant collateral vessel, and a reduction in absolute coronary flow in the donor vessel. Ladwiniec [3] reported the effect of CTO-PCI on donor vessel physiology by measuring coronary pressure and flow velocity before and after PCI and showed that both resting and hyperemic absolute flow volume of the predominant donor vessel decreased, resulting in similar CFR values after PCI. In contrast, a recent study by Stuijfzand, et al. showed that the absolute flow of the PCI target region evaluated by PET significantly increased after CTO-PCI, as did the myocardial flow in the remote area.^2^

### Coronary flow redistribution after revascularization of non-CTO lesions

Limited data are available regarding the effect of non-CTO PCI without visible collateral flow on the coronary flow of remote non-target vessels. A recent study by Aikawa^1^ using PET reported that in 88% of their study population with non-CTO lesions, coronary revascularization improved the regional stress absolute MBF and MFR, whereas no significant change in coronary flow was observed in the remote territories. Another study by Manfrini *et al*. investigated the changes using corrected thrombolysis in myocardial infarction frame count (CTFC) to evaluate the perfusion of myocardial regions supplied by angiographically normal or near-normal coronary arteries after target non-CTO lesion PCI and assess the effect of PCI on coronary flow at rest in sites remote from the target vessel [24]. They concluded that PCI of the target lesion resulted in global improvement by facilitating increased coronary flow at rest to both, the target and remote regions, in patients with stable angina.

CTFC is an established metric to semiquantitatively measure the epicardial blood flow in the absence of significant stenosis and can estimate the degree of impairment in myocardial perfusion [25]; notably, these authors found that CTFCs before and immediately after PCI were similar in the target and non-target arteries. After 6 months, however, the mean CTFC significantly decreased (faster flow) compared with postprocedural CTFCs in both the target and non-target arteries. In the non-target artery, changes in CTFC strongly correlated with pre-PCI CTFC, indicating that the lower the pre-PCI CTFC in the non-target artery, the greater the improvement in epicardial coronary flow at follow-up. The authors interpreted these phenomena as a generalized improvement in microvascular function in both the target and non-target vessels. Our findings are partly in line with these results and strongly suggest the involvement of hyperemic microvascular functional changes in the remote area after PCI.

### Clinical implications

Our serial TDE assessments provide a novel insight into the serial change in coronary physiology of the LAD after successful non-LAD non-CTO PCI and coronary flow redistribution. PCI aims to increase coronary blood flow; however, the present study suggests that non-LAD PCI may decrease both LAD-hDPV and CFVR in more than half of the cases with mild LAD diameter stenosis (33.7% ± 13.4), indicating a reduction in coronary flow capacity, which has been identified as a significant predictor of worse outcomes [26,27]. After PCI of the non-LAD lesion, the hyperemic microvascular resistance in the LAD territory may be affected by epicardial LAD stenosis and potential collateral flow to non-LAD territory, resulting in the redistribution of coronary flow. Complex mechanism may be involved in this response, and further investigation is needed to test our hypothesis generating results and to elucidate if the observed impact on LAD flow is associated with outcomes. Furthermore, the change in flow volume may affect pressure indices currently used for revascularization decision-making. When the functional stenosis severity of LAD is close to the FFR cutoff point before non-LAD PCI, the decision to conduct LAD PCI may change, even though its exact effect on FFR remains to be determined. The prognostic impact of decreased or increased CFR and coronary flow capacity of LAD after non-LAD PCI should also be studied in the future.

### Limitations

This study has several important limitations. First, it included only 50 patients for the final analysis and has inherent limitations in its small study population and nonrandomized, single-center, and observational nature. Second, FFR measurements in LAD were not mandatory in the present study, although FFR was evaluated before and after PCI for non-LAD target vessels. QFR was used as a substitute for FFR in cases without pre-PCI FFR measurements in LAD. Third, in the Doppler echocardiographic examination of LAD coronary flow, pre- and post-PCI LAD flow data are comparable only at identical measurement positions and constant vessel diameters under similar hemodynamics. Nevertheless, a change in the diameter of the LAD after non-LAD PCI or a difference in the functional measurement position cannot be ruled out. Fourth, the post-PCI TDE measurement was performed at 2 days after PCI. Subsequent mid- and long-term changes in coronary flow should be further studied. Fifth, because we only enrolled patients with functionally significant lesions in the RCA or LCx to assess LAD flow after successful non-LAD PCI, it remains to be determined whether our findings can be extrapolated to other coronary arteries. Sixth, the absolute coronary flow volume was not assessed in this study. Seventh, elevated left ventricular end-diastolic pressure (LVEDP) may affect the coronary microcirculation and coronary flow, but no direct LVEDP measurement was performed in this study. Lastly and most importantly, our hyperemic physiological results were obtained under intravenous vasodilator infusion, which indicated that hyperemia was induced in both the target and LAD regions. Different results might have been observed if the intracoronary vasodilator injection is used to induce hyperemia only in the injected vessel territory.

## Conclusion

The hDPV and CFVR of LAD with mild to moderate stenosis decreased after non-CTO and non-LAD PCI. Both the angiographic and physiological stenosis severity of LAD are significant predictors of the change in CFVR after non-LAD PCI.

## Supporting information

S1 Text Supporting information of study methods. (DOCX)
