# Supplementary material for: Early effect of percutaneous coronary intervention of non-left anterior descending artery on coronary flow velocity reserve of left anterior descending artery assessed by transthoracic Doppler echocardiography
Source: PLoS One. 2021 Aug 13;16(8):e0256161. doi: 10.1371/journal.pone.0256161 (PMC8363006; doi:10.1371/journal.pone.0256161)
Supplement: S1 Text — (DOCX) [file pone.0256161.s001.docx]

**Supporting information**

**Percutaneous coronary intervention and FFR measurement**

FFR measurement was performed in all clinically indicated vessels with an angiographically intermediate lesion (40–70%) using a Radi Analyzer Xpress instrument with a single 0.014-inch PressureWire™ (Abbott Vascular, St. Paul, MN, USA). The FFR value was calculated as the ratio of the mean distal coronary pressure to the mean aortic pressure during stable hyperemia, which was induced by intravenous adenosine (140 μg/kg/min through a central vein). After calibration, the wire was advanced and the intracoronary pressure distal to the coronary stenosis was measured. All patients were instructed to strictly refrain from ingesting caffeinated beverages for >24 hours before catheterization. Eligible patients who had non-totally occluded and non-LAD functionally significant stenosis with or without LAD stenosis (<70% stenosis) subsequently underwent preprocedural LAD-CFVR assessment before the scheduled PCI procedure for the non-LAD culprit lesion. No cases with severe LAD stenosis (QCA stenosis >70%) were included, while cases with mild-to-moderate LAD stenosis were intentionally included to explore its relationship with post-PCI LAD physiology.

All patients underwent coronary stent implantation (2^nd^ or 3^rd^ generation drug-eluting stent) with pre-dilatation. The type of stent was selected at the operator’s discretion, and the strategy was determined by the interventionist. To avoid aggressive stent expansion, online quantitative coronary angiography was used to determine the proper stent size. Successful PCI was defined as resulting in <20% residual stenosis, thrombolysis in cases of myocardial infarction grade 3, no side branch occlusion or distal embolization, and no PCI-related myocardial infarction.

**LAD functional assessment using quantitative flow ratio (QFR)**

The functional assessment of the pre-PCI LAD was performed using the QFR. Computation of the QFR was performed offline using offline proprietary software (QAngio XA 3D 2.0.28.0; Medis Medical Imaging Systems B.V., Leiden, Netherlands). First, 2 angiographic images at least 25° apart were transferred to a computer equipped with a QFR system, and three-dimensional (3D) reconstruction of the interrogated vessel was performed with minimal optimization and observer interaction, as previously described [1,2]. The angiographic protocol was optimized for QFR computation. If the angiographic images were not optimal for online QFR computation, another angiographic view was added to obtain an adequate angiographic image. Contrast-flow QFR computation was used in the present study because the contrast-flow model had better diagnostic accuracy as compared to the fixed-flow model[1]. QFR analysis was performed by two well-trained investigators (M.H. and T.S.) who were blinded to the clinical and culprit lesion physiological data. These investigators had obtained the manufacturer’s certificates of completing the training course. The positive functional ischemic threshold was defined as ≤0.80 for the QFR.

**CCTA acquisition**

CT examinations were performed using a 320-slice CT scanner (Aquilion ONE; Canon Medical Systems Corporation, Otawara, Tochigi, Japan). Patients received 20-40 mg of oral metoprolol 1 hour before the scheduled scan if their resting heart rate was >65 beats per minute, and all patients received 0.3 mg of sublingual nitroglycerin just before the scan. Non-contrast cardiac CT images with 3-mm slice thickness were obtained before the coronary CTA to measure coronary artery calcification scores according to the Agatston method [3]. Coronary CTA images were acquired using the following scan protocol: tube voltage=120 kVp; tube current=50–750 mA; gantry rotation speed=350 ms/rotation; field matrix=512 × 512; and scan slice thickness=0.5 mm. As soon as the signal density level in the ascending aorta reached a predefined threshold of 150 Hounsfield units (HU), the acquisition of CT data and an electrocardiogram trace were automatically started. Images were acquired after a bolus injection of 30–60 mL of contrast media (iopamidol; 370 mg iodine/mL; Bayer Yakuhin, Ltd., Osaka, Japan) at a rate of 3-6 mL/s, using prospective ECG-triggering or retrospective ECG-gating with tube current modulation. All scans were performed during a single breath-hold. Images were reconstructed at a window centered at 75% of the R-R interval to coincide with left ventricular (LV) diastasis.

Quantitative assessments of LV mass were performed using the Aquarius iNtuition Workstation Edition version 4.4.13 (TeraRecon Inc., Foster City, CA, USA). The cardiac mass was calculated as the left ventricular myocardial volume derived by manually corrected and automated delineation of the epicardial and endocardial contours, which was multiplied by the specific gravity of myocardial tissue. Papillary muscles were not included in the calculation of LV mass. The target lesion location was identified on a 3D volume-rendered coronary CTA image by the analysts of the subtended cardiac mass using angiographic guidance and FFR values. Coronary artery-based myocardial segmentation was performed to evaluate a coronary lesion-specific cardiac mass using the same dedicated software (Aquarius iNtuition Edition version 4.4.13; TeraRecon Inc.) by an expert investigator who was blinded to the clinical, angiographic, and physiological data [4].

**Supplemental references**

1. Tu S, Barbato E, Koszegi Z, Yang J, Sun Z, Holm NR, et al. Fractional flow reserve calculation from 3-dimensional quantitative coronary angiography and TIMI frame count: a fast computer model to quantify the functional significance of moderately obstructed coronary arteries. JACC Cardiovasc Interv. 2014;7(7):768-77.

2. Tu S, Westra J, Yang J, von Birgelen C, Ferrara A, Pellicano M, et al. Diagnostic Accuracy of Fast Computational Approaches to Derive Fractional Flow Reserve From Diagnostic Coronary Angiography: The International Multicenter FAVOR Pilot Study. JACC Cardiovasc Interv. 2016;9(19):2024-35.

3. Agatston AS, Janowitz WR, Hildner FJ, Zusmer NR, Viamonte M, Jr., Detrano R. Quantification of coronary artery calcium using ultrafast computed tomography. J Am Coll Cardiol. 1990;15(4):827-32.

4. Kang SJ, Yang DH, Kweon J, Kim YH, Lee JG, Jung J, et al. Better Diagnosis of Functionally Significant Intermediate Sized Narrowings Using Intravascular Ultrasound-Minimal Lumen Area and Coronary Computed Tomographic Angiography-Based Myocardial Segmentation. Am J Cardiol. 2016;117(8):1282-8.
